# The Genetics of Alcohol and Other Drug Dependence

**Published:** 2008

**Authors:** Danielle M. Dick, Arpana Agrawal

**Keywords:** Alcohol and other drug (AOD) dependence (AODD), co-morbid AOD dependence, genetics and heredity, genetic theory of AODD, genetic risk factors, AODR genetic markers

## Abstract

Alcohol dependence and dependence on other drugs frequently co-occur, and strong evidence suggests that both disorders are, at least in part, influenced by genetic factors. Indeed, studies using twins suggest that the overlap between dependence on alcohol and on other drugs largely results from shared genetic factors. This common genetic liability, which also extends to antisocial behavior, has been conceptualized as a general predisposition toward a variety of forms of psychopathology characterized by disinhibited behavior (i.e., externalizing psychopathology). Accordingly, many of the genetic factors affecting risk for dependence on alcohol or other drugs appear to act through a general externalizing factor; however, other genetic factors appear to be specific to a certain disorder. In recent years, researchers have identified numerous genes as affecting risk for dependence on alcohol and other drugs. These include genes involved in alcohol metabolism as well as in the transmission of nerve cell signals and modulation of nerve cell activity (i.e., γ-aminobutyric acid [GABA] and acetylcholinergic neurotransmission and the endogenous opioid and cannabinoid systems).

This article explores the hypothesis that certain genetic factors increase a person’s risk of both alcohol abuse and dependence and other drug abuse and dependence. It first reviews the evidence suggesting that certain genetic factors contribute to the development of alcohol and other drug (AOD) use disorders, as well as to the development of a variety of forms of externalizing psychopathology—that is, psychiatric disorders characterized by disinhibited behavior, such as antisocial personality disorder, attention deficit/hyperactivity disorder, and conduct disorder. After summarizing the difficulties associated with, and recent progress made in, the identification of specific genes associated with AOD dependence, the article then discusses evidence that implicates several genes in a person’s risk for dependence on both alcohol and illicit drugs.

## Genetic Epidemiology of AOD Dependence

Alcohol dependence frequently co-occurs with dependence on illicit drugs (Hasin et al. 2007). Both alcohol use disorders (i.e., alcohol abuse and alcohol dependence) and drug use disorders (drug abuse and drug dependence) are influenced by several factors. For example, family, twin, and adoption studies^1^ have convincingly demonstrated that genes contribute to the development of alcohol dependence, with heritability estimates ranging from 50 to 60 percent for both men and women (McGue 1999). Dependence on illicit drugs only more recently has been investigated in twin samples, but several studies now suggest that illicit drug abuse and dependence also are under significant genetic influence. In these studies of adult samples, heritability estimates ranged from 45 to 79 percent (for reviews, see Agrawal and Lynskey 2006; Kendler et al. 2003*a*; Tsuang et al. 2001).

Twin studies also can be used to assess the extent to which the *co-occurrence* of disorders is influenced by genetic and/or environmental factors. Thus, a finding that the correlation between alcohol dependence in twin 1 and drug dependence in twin 2 is higher for identical (i.e., monozygotic) twins, who share 100 percent of their genes, than for fraternal (i.e., dizygotic) twins, who share on average only 50 percent of their genes, indicates that shared genes influence the risk of both alcohol and drug dependence. The twin studies conducted to date support the role of such shared genetic factors. For example, in the largest twin study of the factors underlying psychiatric disorders, Kendler and colleagues (2003*b*) analyzed data from the Virginia Twin Registry and found that a common genetic factor contributed to the total variance in alcohol dependence, illicit drug abuse and dependence, conduct disorder, and adult antisocial behavior. This pattern also has been identified in several other independent twin studies (Krueger et al. 2002; Young et al. 2000). Taken together, these findings suggest that a significant portion of the genetic influence on alcohol dependence and drug dependence is through a general predisposition toward externalizing disorders, which may manifest in different ways (e.g., different forms of AOD dependence and/or antisocial behavior) (see figure). However, some evidence also suggests that disorder-specific genetic influences contribute to AOD dependence (Kendler et al. 2003*b*). These specific influences likely reflect the actions of genes that are involved in the metabolism of individual drugs.

The idea that alcohol and drug dependence share a genetic liability with each other, as well as with other forms of externalizing psychopathology, is further supported by electrophysiological studies recording the brain’s electrical activity. These studies, which are conducted using electrodes placed on the person’s scalp, provide a noninvasive, sensitive method of measuring brain function in humans. They generate a predictable pattern in the height (i.e., amplitude) and rate (i.e., frequency) of brain waves that can show characteristic abnormalities in people with certain types of brain dysfunction. For example, electrophysiological abnormalities have been observed in people with a variety of externalizing disorders as well as in unaffected children of these people. These findings suggest that electrophysiological measurements can be used as markers of a genetic vulnerability to externalizing disorders.

One commonly measured electrophysiological characteristic is the so-called P3 component of an event-related potential—that is, a spike in brain activity that occurs about 300 milliseconds after a person is exposed to a sudden stimulus (e.g., a sound or light). Researchers have observed that the amplitude of the P3 component is reduced in alcohol-dependent people and their children, suggesting that this abnormality is a marker for a genetic predisposition to alcohol dependence (Porjesz et al. 1995). However, the abnormal P3 response is not specific to alcohol dependence but appears to be associated with a variety of disinhibitory disorders, including other forms of drug dependence, childhood externalizing disorders, and adult antisocial personality disorder, again suggesting a shared underlying predisposition to multiple forms of AOD dependence and other externalizing problems (Hicks et al. 2007).^2^

Interestingly, electrophysiological abnormalities are most pronounced in alcohol-dependent people who also have a diagnosis of illicit drug abuse or dependence (Malone et al. 2001). This observation is consistent with data from twin and family studies suggesting that co-morbid dependence on alcohol and another drug represents a more severe disorder with higher heritability than dependence on one drug alone (Johnson et al. 1996; Pickens et al. 1995). This conclusion also appears to be supported by new studies exploring the roles of specific genes, which are discussed later in this article.

## Identifying Specific Genes Related to AOD Dependence

With robust evidence indicating that genes influence both alcohol dependence and dependence on illicit drugs, efforts now are underway to identify specific genes involved in the development of these disorders. This identification, however, is complicated by many factors. For example, numerous genes are thought to contribute to a person’s susceptibility to alcohol and/or drug dependence, and affected people may carry different combinations of those genes. Additionally, environmental influences have an impact on substance use, as does gene– environment interaction (Heath et al. 2002). Finally, the manifestation of AOD dependence varies greatly among affected people, for example, with respect to age of onset of problems, types of symptoms exhibited (i.e., symptomatic profile), substance use history, and presence of co-morbid disorders.

Despite the complications mentioned above, the rapid growth in research technologies for gene identification in recent years has led to a concomitant increase in exciting results. After suffering many disappointments in early attempts to identify genes involved in complex behavioral outcomes (i.e., phenotypes), researchers now are frequently succeeding in identifying genes that help determine a variety of clinical phenotypes. These advances have been made possible by several factors. First, advances in technologies to identify a person’s genetic makeup (i.e., genotyping technology) have dramatically lowered the cost of genotyping, allowing for high-throughput analyses of the entire genome. Second, the completion of several large-scale research endeavors, such as the Human Genome Project, the International HapMap Project,^3^ and other government and privately funded efforts, have made a wealth of information on variations in the human genome publicly available. Third, these developments have been complemented by advances in the statistical analysis of genetic data.

Several large collaborative projects that strive to identify genes involved in AOD dependence currently are underway. The first large-scale project aimed at identifying genes contributing to alcohol dependence was the National Institute on Alcohol Abuse and Alcoholism (NIAAA)-sponsored Collaborative Study on the Genetics of Alcoholism (COGA), which was initiated in 1989. This study, which involves collaboration of investigators at several sites in the United States, examines families with several alcohol-dependent members who were recruited from treatment centers across the United States. This study has been joined by several other gene identification studies focusing on families affected with alcohol dependence, including the following:

- A sample of Southwestern American Indians (Long et al. 1998);
- The Irish Affected Sib Pair Study of Alcohol Dependence (Prescott et al. 2005*a*);
- A population of Mission Indians (Ehlers et al. 2004);
- A sample of densely affected families collected in the Pittsburgh area (Hill et al. 2004); and
- An ongoing data collection from alcohol-dependent individuals in Australia.

Importantly, most of these projects include comprehensive psychiatric interviews that focus not only on alcohol use and alcohol use disorders but which also allow researchers to collect information about other drug use and dependence. This comprehensive approach permits researchers to address questions about the nature of genetic influences on AOD dependence, as discussed below.

More recently, additional studies have been initiated that specifically seek to identify genes contributing to various forms of illicit drug dependence as well as general drug use problems (for more information, see http://www.nida.nih.gov/about/organization/Genetics/consortium/index.html). Through these combined approaches, researchers should be able to identify both genes with drug-specific effects and genes with more general effects on drug use. The following sections focus on several groups of genes that have been identified by these research efforts and which have been implicated in affecting risk for dependence on both alcohol and illicit drugs.

### Genes Encoding Proteins Involved in Alcohol Metabolism

The genes that have been associated with alcohol dependence most consistently are those encoding the enzymes that metabolize alcohol (chemically known as ethanol). The main pathway of alcohol metabolism involves two steps. In the first step, ethanol is converted into the toxic intermediate acetaldehyde; this step is mediated by the alcohol dehydrogenase (ADH) enzymes. In a second step, the acetaldehyde is further broken down into acetate and water by the actions of aldehyde dehydrogenase (ALDH) enzymes. The genes that encode the ADH and ALDH enzymes exist in several variants (i.e., alleles) that are characterized by variations (i.e., polymorphisms) in the sequence of the DNA building blocks. One important group of ADH enzymes are the ADH class I isozymes ADH1A, ADH1B, and ADH1C. For both the genes encoding ADH1B and those encoding ADH1C, several alleles resulting in altered proteins have been identified, and the proteins encoded by some of these alleles exhibit particularly high enzymatic activity in laboratory experiments (i.e., in vitro) (Edenberg 2007). This suggests that in people carrying these alleles, ethanol is more rapidly converted to acetaldehyde.^4^ Several studies have reported lower frequencies of both the ADH1B*2 and ADH1C*1 alleles, which encode some of the more active proteins, among alcoholics than among non-alcoholics in a variety of East Asian populations (e.g., Shen et al. 1997) and, more recently, in European populations (Neumark et al. 1998; Whitfield et al. 1998).

In addition, genome-wide screens to identify genes linked to alcoholism and alcohol-related traits have been conducted in three independent samples consisting largely of people of European descent—the COGA study (Saccone et al. 2000), the Irish Affected Sib Pair Study of Alcohol Dependence (Prescott et al. 2005*a*), and an Australian sample (Birley et al. 2005). These studies have found evidence that a region on chromosome 4 containing the ADH gene cluster shows linkage to the phenotypes studied. This cluster contains, in addition to the genes encoding ADH class I isozymes, the genes *ADH4*, *ADH5*, *ADH6*, and *ADH7*, which encode other ADH enzymes. Polymorphisms exist for each of these genes, some of which also have been associated with alcohol dependence (Edenberg et al. 2006; Luo et al. 2006*a*,*b*; Prescott et al. 2005*b*).

Interestingly, the effects of these genes do not appear to be limited to alcohol dependence. One study compared the frequency of alleles that differed in only one DNA building block (i.e., single nucleotide polymorphisms [SNPs]) throughout the genome between people with histories of illicit drug use and/or dependence and unrelated control participants. This study detected a significant difference for a SNP located near the ADH gene cluster (Uhl et al. 2001). More recent evidence suggests that genetic variants in the *ADH1A*, *ADH1B*, *ADH1C*, *ADH5*, *ADH6*, and *ADH7* genes are associated with illicit drug dependence and that this association is not purely attributable to co-morbid alcohol dependence (Luo et al. 2007). The mechanism by which these genes may affect risk for illicit drug dependence is not entirely clear. However, other observations^5^ also indicate that enzymes involved in alcohol metabolism may contribute to illicit drug dependence via pathways that currently are unknown but independent of alcohol metabolism (Luo et al. 2007).

### Genes Encoding Proteins Involved in Neurotransmission

AODs exert their behavioral effects in part by altering the transmission of signals among nerve cells (i.e., neurons) in the brain. This transmission is mediated by chemical messengers (i.e., neurotransmitters) that are released by the signal-emitting neuron and bind to specific proteins (i.e., receptors) on the signal-receiving neuron. AODs influence the activities of several neurotransmitter systems, including those involving the neurotransmitters γ-aminobutyric acid (GABA), dopamine, and acetylcholine, as well as naturally produced compounds that structurally resemble opioids and cannabinoids. Accordingly, certain genes encoding components of these neurotransmitter systems may contribute to the risk of both alcohol dependence and illicit drug dependence.

#### Genes Encoding the GABA_A_ Receptor

GABA is the major inhibitory neurotransmitter in the human central nervous system—that is, it affects neurons in a way that reduces their activity. Several lines of evidence suggest that GABA is involved in many of the behavioral effects of alcohol, including motor incoordination, anxiety reduction (i.e., anxiolysis), sedation, withdrawal signs, and preference for alcohol (Grobin et al. 1998). GABA interacts with several receptors, and much of the research on alcohol’s interactions with the GABA system has focused on the GABA_A_ receptor. This receptor also is the site of action for several medications that frequently are misused and have high addictive potential, such as benzodiazepines, barbiturates, opiates, α-hydroxybutyrates, and other sedative–hypnotic compounds. Accordingly, this receptor likely is involved in dependence on these drugs as well (Orser 2006).

The GABA_A_ receptor is composed of five subunits that are encoded by numerous genes, most of which are located in clusters. Thus, chromosome 4 contains a cluster comprising the genes *GABRA2*, *GABRA4*, *GABRB1*, and *GABRG1*; chromosome 5 contains *GABRA1, GABRA6, GABRB2*, and *GABRG2*; and chromosome 15 contains *GABRA5, GABRB3*, and *GABRG3* (see http://www.ncbi.nlm.nih.gov/sites/entrez?db=gene).

Interest in the GABA_A_ receptor genes on chromosome 4 grew when this region consistently was identified in genome-wide scans looking for linkage with alcohol dependence (Long et al. 1998; Williams et al. 1999). Subsequently, COGA investigators systematically evaluated short DNA segments of known location (i.e., genetic markers) that were situated in the GABA_A_ receptor gene cluster on chromosome 4. These studies found that a significant association existed between multiple SNPs in the *GABRA2* gene and alcohol dependence (Edenberg et al. 2004). This association has been replicated in multiple independent samples (Covault et al. 2004; Fehr et al. 2006; Lappalainen et al. 2005; Soyka 2007). In addition, the same SNPs in the *GABRA2* gene have been shown to be associated with drug dependence in both adults and adolescents (Dick et al. 2006*a*), as well as with the use of multiple drugs in another independent sample (Drgon et al. 2006).

Variations in the *GABRA2* gene are associated not only with AOD dependence but also with certain electrophysiological characteristics (i.e., endophenotypes) in the COGA sample (Edenberg et al. 2004). As reviewed above, these electrophysiological characteristics are not unique to alcohol dependence but also are found in individuals with other forms of externalizing psychopathology. This association supports the hypothesis that the *GABRA2* gene generally is involved in AOD use and/or externalizing problems. Interestingly, subsequent analyses investigating the role of *GABRA2* in drug dependence (Agrawal et al. 2006) found that the association with *GABRA2* was strongest in people with co-morbid AOD dependence, with no evidence of association in people who were only alcohol dependent. This observation supports the assertion that co-morbid AOD dependence may represent a more severe, genetically influenced form of the disorder.

Several other GABA_A_ receptor genes have yielded more modest evidence of association with different aspects of AOD dependence. Thus, *GABRB3* (Noble et al. 1998) and *GABRG3* (Dick et al. 2004) are modestly associated with alcohol dependence, *GABRA1* (Dick et al. 2006*b*) is associated with alcohol-related phenotypes (e.g., history of alcohol-induced blackouts and age at first drunkenness), and *GABRG2* (Loh et al. 2007) is associated with aspects of drug dependence. These findings await confirmation in independent samples.

#### Genes Involved in the Cholinergic System

The cholinergic system includes neurons that either release the neurotransmitter acetylcholine or respond to it. Acetylcholine generally has excitatory effects in the human central nervous system—that is, it affects neurons in a way that enhances their activity. It is thought to be involved in such processes as arousal, reward, learning, and short-term memory. One of the receptors through which acetylcholine acts is encoded by a gene called *CHRM2*. In the COGA sample, linkage was observed between a region on chromosome 7 that contains the *CHRM2* gene and alcohol dependence, and subsequent experiments confirmed that an association existed between alcohol dependence and the *CHRM2* gene (Wang et al. 2004). This association has been replicated in a large independent study (Luo et al. 2005) that also found evidence that the gene was associated with drug dependence.

As with the *GABRA2* gene described above, the association between *CHRM2* and alcohol dependence in the COGA sample was strongest in people who had co-morbid AOD dependence (Dick et al. 2007). Additional analyses in the COGA sample have suggested that *CHRM2* is associated with a generally increased risk of externalizing disorders, including symptoms of alcohol dependence and drug dependence (Dick et al. 2008). This potential role of *CHRM2* in contributing to the general liability of AOD use and externalizing disorders is further supported by findings that *CHRM2*, like *GABRA2*, also is associated with certain electrophysiological endophenotypes (Jones et al. 2004).

#### Genes Involved in the Endogenous Opioid System

Endogenous opioids are small molecules naturally produced in the body that have similar effects as the opiates (e.g., morphine and heroin) and which, among other functions, modulate the actions of other neurotransmitters. The endogenous opioid system has been implicated in contributing to the reinforcing effects of several drugs of abuse, including alcohol, opiates, and cocaine. This is supported by the finding that the medication naltrexone, which prevents the normal actions of endogenous opioids (i.e., is an opioid antagonist), is useful in the treatment of alcohol dependence and can reduce the number of drinking days, amount of alcohol consumed, and risk of relapse.

Research on the role of the endogenous opioids in AOD dependence has centered mainly on a gene called *OPRM1*, which encodes one type of opioid receptor (i.e., the μ-opioid receptor), although the results so far have been equivocal. This gene contains a polymorphism resulting in a different protein product (i.e., a non-synonymous polymorphism) that in one study was found to bind one of the endogenous opioids (i.e., β-endorphin) three times as strongly as the main variant of the gene (Bond et al. 1998); other studies, however, could not confirm this finding (Befort et al. 2001; Beyer et al. 2004).

Laboratory studies have suggested that *OPRM1* is associated with sensitivity to the effects of alcohol (Ray and Hutchison 2004). In addition, several studies have reported evidence of an association between *OPRM1* and drug dependence (e.g., Bart et al. 2005). Other studies, however, have failed to find such an association (e.g., Bergen et al. 1997), and a combined analysis of several studies (i.e., a meta-analysis) concluded that no association exists between the most commonly studied *OPRM1* polymorphism and drug dependence (Arias et al. 2006). However, this finding does not preclude the possibility that other genetic variants in *OPRM1* and/or other genes related to the endogenous opioid system are involved in risk for drug dependence. For example, a recent study determining the genotypes of multiple genetic variants across the gene uncovered evidence of association with *OPRM1* and AOD dependence (Zhang et al. 2006).

Researchers also have investigated genetic variations in other opioid receptors and other components of the endogenous opioid system; however, the results have been mixed. One study (Zhang et al. 2007) found modest support that the genes *OPRK1* and *OPRD1*—which encode the κ and δ-opioid receptors, respectively—are associated with some aspects of drug dependence. Other researchers (Xuei et al. 2007) reported evidence that the genes *PDYN*, *PENK*, and *POMC*—which encode small molecules (i.e., peptides) that also bind to opioid receptors—may be associated with various aspects of drug dependence.

#### Genes Involved in the Endogenous Cannabinoid System

Endogenous cannabinoids are compounds naturally produced in the body that have a similar structure to the psychoactive compounds found in the cannabis plant and which bind cannabinoid receptors. The endogenous cannabinoid system is thought to regulate brain circuits using the neurotransmitter dopamine, which likely helps mediate the rewarding experiences associated with addictive substances. The main cannabinoid receptor in the brain is called CB1 and is encoded by the *CNR1* gene, which is located on chromosome 6. This gene is an excellent candidate gene for being associated with AOD dependence because the receptor encoded by this gene is crucial for generating the rewarding effects of the compound responsible for the psychoactive effects associated with cannabis use (i.e., Δ9-tetrahydrocannabinol). However, the findings regarding the association between *CNR1* and AOD dependence to date have been equivocal, with some studies producing positive results (e.g., Zhang et al. 2004) and others producing negative results (e.g., Herman et al. 2006). Most recently, Hopfer and colleagues (2006) found that a SNP in the *CNR1* gene was associated with cannabis dependence symptoms. ^6^ Moreover, this SNP was part of several sets of multiple alleles that are transmitted jointly (i.e., haplotypes), some of which are associated with developing fewer dependence symptoms, whereas others are associated with an increased risk for cannabis dependence. Finally, a recent case–control study found that multiple genetic variants in *CNR1* were significantly associated with alcohol dependence and/or drug dependence (Zuo et al. 2007).

## Conclusions

For both alcohol dependence and drug dependence, considerable evidence suggests that genetic factors influence the risk of these disorders, with heritability estimates of 50 percent and higher. Moreover, twin studies and studies of electrophysiological characteristics indicate that the risk of developing AOD dependence, as well as other disinhibitory disorders (e.g., antisocial behavior), is determined at least in part by shared genetic factors. These observations suggest that some of a person’s liability for AOD dependence will result from a general externalizing factor and some will result from genetic factors that are more disorder specific.

Several genes have been identified that confer risk to AOD dependence. Some of these genes—such as *GABRA2* and *CHRM2—* apparently act through a general externalizing phenotype. For other genes that appear to confer risk of AOD dependence—such as genes involved in alcohol metabolism and in the endogenous opioid and cannabinoid systems—however, the pathways through which they affect risk remain to be elucidated. Most of the genes reviewed in this article originally were found to be associated with alcohol dependence and only subsequently was their association with risk for dependence on other illicit drugs discovered as well. Furthermore, studies that primarily aim to identify genes involved in dependence on certain types of drugs may identify different variants affecting risk, underscoring the challenge of understanding genetic susceptibility to different classes of drugs.

This review does not exhaustively cover all genes that to date have been implicated in alcohol and illicit drug dependence. For example, several genes encoding receptors for the neurotransmitter dopamine have been suggested to determine at least in part a person’s susceptibility to various forms of drug dependence. In particular, the *DRD2* gene has been associated with alcohol dependence (Blum et al. 1990) and, more broadly, with various forms of addiction (Blum et al. 1996). This association remains controversial, however, and more recent studies suggest that the observed association actually may not involve variants in the *DRD2* gene but variants in a neighboring gene called *ANKK1* (Dick et al. 2007*b*). Studies to identify candidate genes that influence dependence on illicit drugs, but not on alcohol, are particularly challenging because of the high co-morbidity between alcohol dependence and dependence on illicit drugs. Therefore, meaningful studies require large sample sizes to include enough drug-dependent people with no prior history of alcohol dependence.

The increasingly rapid pace of genetic discovery also has resulted in the identification of several genes encoding other types of proteins that appear to be associated with alcohol use and/or dependence. These include, for example, two genes encoding taste receptors (i.e., the *TAS2R16* gene [Hinrichs et al. 2006] and the *TAS2R38* gene [Wang et al. 2007]) and a human gene labeled *ZNF699* (Riley et al. 2006) that is related to a gene previously identified in the fruit fly *Drosophila* as contributing to the development of tolerance to alcohol in the flies. Future research will be necessary to elucidate the pathways by which these genes influence alcohol dependence and/or whether they are more broadly involved in other forms of drug dependence.

## Figures and Tables

**Figure:**
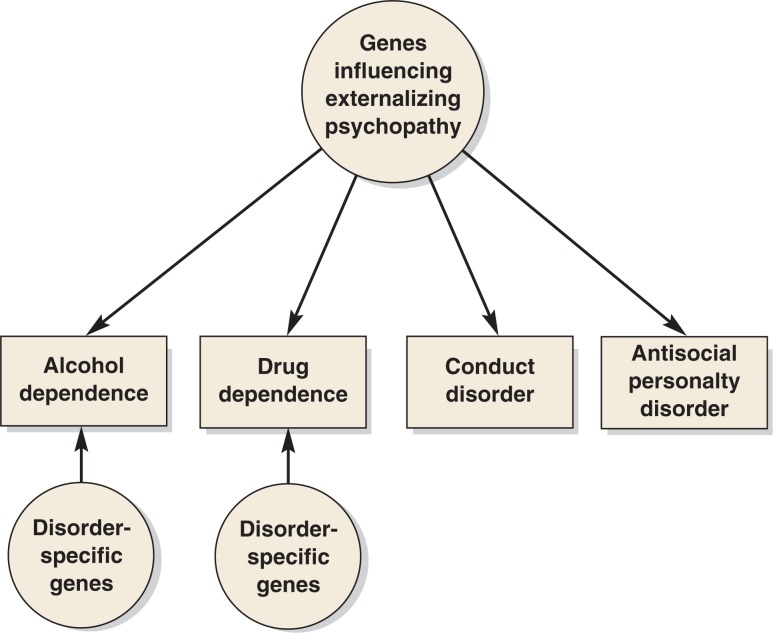
Schematic representation of a model to illustrate the influence of genetic factors on the development of alcohol dependence, dependence on other drugs, and other externalizing disorders (e.g., conduct disorder or antisocial personality disorder). Some of the proposed genetic factors are thought to have a general influence on all types of externalizing conditions, whereas others are thought to have a disorder-specific influence.

## Footnotes

1 For a definition of these and other terms, see the glossary, pp. 177–179.

2 Abnormalities in the P3 response also have been associated with risk for other psychiatric disorders, such as schizophrenia (van der Stelt et al. 2004).

3 The International HapMap Project is a multicountry effort to identify and catalog genetic similarities and differences in human beings by comparing the genetic sequences of different individuals in order to identify chromosomal regions where genetic variants are shared. Using the information obtained in the HapMap Project, researchers will be able to find genes that affect health, disease, and individual responses to medications and environmental factors.

4 Rapid acetaldehyde production can lead to acetaldehyde accumulation in the body, which results in highly unpleasant effects, such as nausea, flushing, and rapid heartbeat, that may deter people from drinking more alcohol.

5 For example, the medication disulfiram, which inhibits another enzyme involved in alcohol metabolism called aldehyde dehydrogenase 2 (ALDH2) and is used for treatment of alcoholism, has demonstrated a treatment effect in cocaine dependence (Luo et al. 2007).

6 The SNP was not located in one of those gene regions that encode the actual receptor (i.e., in an exon) but in a region that is part of the gene but is eliminated during the process of converting the genetic information into a protein product (i.e., in an intron).
